# Integration of Phase Change Material in the Design of Solar Concentrator-Based Water Heating System

**DOI:** 10.3390/e24010057

**Published:** 2021-12-28

**Authors:** Teuku Azuar Rizal, Muhammad Amin, Syamsul Bahri Widodo, Nazaruddin Abdul Rachman, Fazri Amir, Nurhalim Pane, Teuku Meurah Indra Mahlia

**Affiliations:** 1 Department of Mechanical Engineering, Universitas Samudra, Kota Langsa 24416, Indonesia; azuarrizal@unsam.ac.id (T.A.R.); syamsulbahri@unsam.ac.id (S.B.W.); nazaruddin@unsam.ac.id (N.A.R.); fazri@unsam.ac.id (F.A.); nurhalimpane@gmail.com (N.P.); 2 Centre for Technology in Water and Wastewater, School of Civil and Environmental Engineering, University of Technology Sydney, Ultimo, NSW 2007, Australia

**Keywords:** phase change materials, solar concentrator, cylindrical trough collector, water heating system, thermal efficiency

## Abstract

Indonesia has been blessed with excellent solar heat distribution, which can be used as renewable energy to heat water. Various technologies have been developed to utilize these inexhaustible thermodynamic resources, in the form of photons arrays, converted into concentrated heat for daily use, i.e., solar water heater. This renewable-based water heating system can provide significant energy efficiency, benefit the environment, and reduce energy use costs. This experimental study attempts to harvest the energy from the sun using a cylindrical through collector (CTC) type solar concentrator. The CTC was made of the solar reflective film (SRF) affixed to concentrator collector surfaces which was then mounted on an adjustable angle frame of the concentrator collector support. The heat generated from the concentrator was stored in water, and phase change material is embedded in the system to retain the heat longer. The research was carried out in Langsa City, Aceh, Indonesia. The results showed that water heaters using CTC systems could produce 16 L of hot water retained at 40–60 °C for four hours. With the addition of beeswax, the water temperature of the same capacity can be maintained at 40–60 °C for around 5 h. This technology demonstrated an excellent result that produces as much as 60 L of water per day, increasing solar thermal energy efficiency. This technology presented a great potential for replication or even for further development on an industrial scale.

## 1. Introduction

Indonesia has been blessed with excellent solar heat distribution. Unlike fossil fuels, solar energy has become a prominent inexhaustible thermodynamic resource and will prevail as a competitive resource in the future [1,2]. Although the development of solar water heaters in Indonesia has not been as popular and rapid as in China [3,4], some local products have played a small role in the market. Furthermore, this technology can absorb solar energy as a fuel to heat water and reduce harmful emissions simultaneously [3]. The selection of a sound water heating system can provide significant energy efficiency, benefit the environment, and minimize energy-use costs [4]. Solar water have been gaining rapid momentum as an environmentally friendly tool in their application as a renewable energy source, and to improve the efficiency and effectiveness of its energy conversion, solar energy is absorbed using solar collectors. Several previous studies have also tested the efficiency of test kits under the influence of concentrator collector forms. Research was conducted during sunrise and successfully increased water temperature to 53 °C with the initial temperature of the water being 24 °C [5]. Similar studies used a mini parabolic shape, operated between 09.30–11.00 WIB, and successfully increased the water temperature from 34 °C to 54 °C with pressure 1.4 bar [6].

The heat generated from the concentrator is stored in water, and Phase Change Material (PCM) is used to retain that heat. This process reduces the mismatch between supply and energy demand and increases the efficiency of solar thermal energy technology. PCM acts as a storage container for heat energy obtained in energy absorbed/generated when there is a phase change from liquid to gas, liquid to solid, or vice versa. Heat is stored inside the PCM during the highest latent heat storage point (off-Peak) and is gradually released during the peak period, and this has been proven to reduce the cost of consuming conventional electric heating energy in heating and DHW systems [7].

This study aims to create a solar water heater using solar collector technology with a concentrator system equipped with a beeswax-based PCM to maintain thermal performance in heated water. The focus of the study relates to solar concentrators using PCM.

According to the Indonesian Government’s Department of Minerals and Energy Resources, the average daily insolation in Indonesia is about 4.8 kWh/m^2^ with variations of 9% to 10% and radiates for 10–12 h per day. The absorbed energy is then returned to nature by about 30%, 47% is converted into heat energy, and about 23% is used for working circulation above the Earth’s surface, as well as 0.25% received by wind, waves, currents, and 0.025% is involved in photosynthesis in plants [8,9]. 

In general, several technologies have been developed to utilize insolation optimally, i.e., through solar collectors, solar *cells,* and technology that convert solar into valuable energy, such as heat or electricity. The most widely studied subject is solar heat technology, which is solar water heaters [10]. A solar cell can produce a current of around 20 mA and can produce a constant voltage of around 0.5–0.7-volts in times of moderate sunny weather, and if the position of the solar cell is perpendicular and to obtain optimal results, it can be determined from the construction of the solar cell itself. It can be concluded that solar cells can produce a power of 0.6 V x 20 mA = 12 mW [11].

As solar technology continues to develop, solar water heaters and solar collectors have been the most prominent approach for further advancements. The solar water heater is one of the tools to collect heat from solar energy used to heat water. This water heating uses solar collectors as its main component. Solar collectors convert insolation into heat energy. The primary function of a solar collector is to absorb insolation and pass that energy on the fluid it passes through the collector. The temperature generated from this solar water heater is about 55–65°C [4,12,13,14].

Recently, there has been progress related to the integration of PCMs in solar collector has been reported, which covers (i) passive solar collector (PSC), (ii) evacuated tube solar collectors (ETSC), and (iii) concentrated type solar collector (CTSC)

PSC can absorb solar heat and convert it into natural heat energy, for example, glass can absorb heat and store heat, or heat from the outside of the wall that is heated by the sun, then the heat gradually moves through the inner surface of the wall, and heats the indoor room. [15]. Sun and Wang [16] developed a new passive solar collector-storage wall system with PCMs, which assists in energy conservation and significantly improves indoor thermal comfort. 

**Figure 1:**
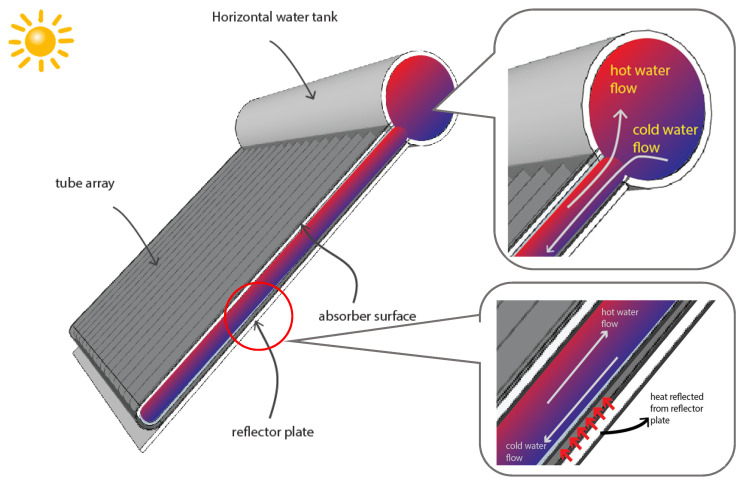
Illustration of cross-sectional view of collector’s evacuated tube in solar water heater application.

The ETSC type consists of an evacuated glass tube. Each tube contains a thin plate that can absorb heat and is glued to the pipes in the glass tube, where the vacuum process takes place to avert heat loss. An example of an evacuated tube collector scheme is presented in Figure 1, and for this particular condition, the water temperature inside can reach 52–62 °C [17]. Kumar et al. [18] analyzed the impacts of nanofluids on the performance of ETSC. Structural modifications by integrating PCM into the system have improved the overall thermal performance. The system is applicable for poly-generation applications of energy. Essa et al. [19] experimentally studied the ETSC with a helically finned heat pipe using PCMs. Two collectors were used, namely the control system and the type with helical fins. The experiments accommodated for different flow rates. The helical fines achieved higher peak temperatures than the conventional at high flow rates. 

**Figure 2:**
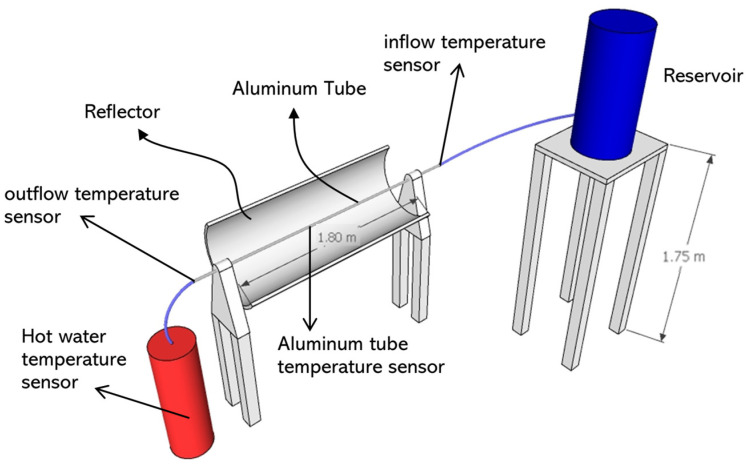
Illustration of a model of concentrated type collector developed in previous research.

Fatmawati et al. [20] experimented with the concentrated type of collector that captures insolation using a mirror or Fresnel lens, where the heat is focused on pipes fed by liquid. Fresnel lenses are added to reflectors and connected using a mechanical system so that they can follow the sun’s heat. This collector can generate more heat than the flat plate collector, but it is costly to make and very complicated to operate. An example of a concentrated collector is illustrated in Figure 2. The result shows that the maximum insolation at 326 W/m^2^ was obtained at 13.00 WIB, which increased the collector temperature to 50 °C and the water temperature in the container to 40 °C. Minimum insolation is 186 W/m^2,^ which occurs at 10:00 WIB, which raises the collector temperature to 39 °C from its initial temperature of 35 °C. The highest conduction heat-loss rate was 29 W, while the lowest was 16.86 W. 

**Figure 3:**
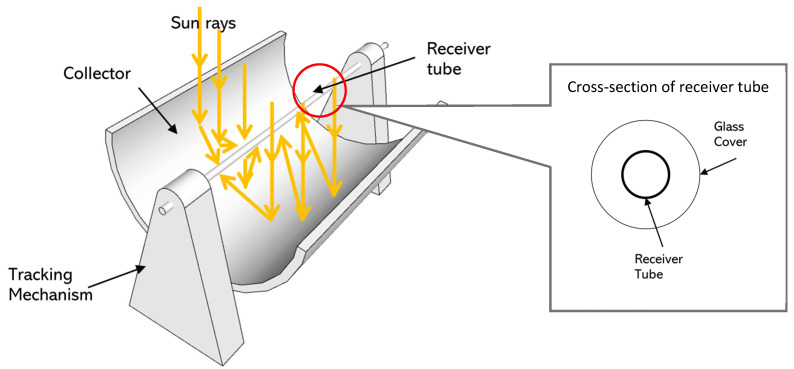
Illustration of sun radiation focused onto receiver tube.

The concentrated collector in the experiment was equipped with concave Fresnel to facilitate radiation to focus the insolation onto a copper pipe mounted in the middle of the device. The lens captured light from all sides due to its concave shape and sent it to a focal point to gain maximum heat [14]. If the Fresnel is smaller than the radius of its curvature, then the reflected light only forms a slight angle when reflected on the cheek in the middle, as in Figure 3. 

Maulana et al. [5] built a test tool to understand the effect of concentrate collector forms on the efficiency of solar water heaters. The test instruments are a cylindrical trough collector (CTC) and parabolic trough collector (PTC). 

The study was conducted in tropical climates from morning to evening, with initial temperatures of 24 °C in the morning and 27 °C in the afternoon. At 13.0 WIB, the temperature of pipes in CTC collectors reached 54 °C, while PTC collectors only 53 °C. At 13.30 WIB–18.0 WIB, the temperature of pipe surfaces in collectors continued to decrease due to the intensity of insolation received by collectors is decreasing. 

**Figure 4:**
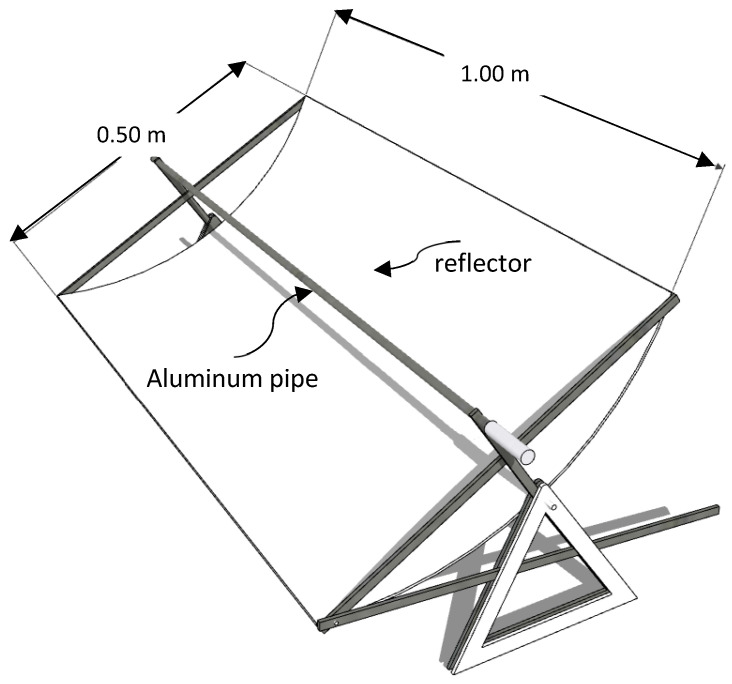
Sample of mini parabolic trough collector design.

In another study conducted by Prastika and Munir [6], PTC was made using various materials and sizes to find the most efficient design. The location where PTC is operated also determines the results of total heating, and the material used also affected the result; therefore, the shiny zinc material was chosen as a reflector, and an aluminum pipe without a glass sheath acted as an absorber. With a relatively small size (1 m long and aperture width of 0.5 m), PTC can produce the highest temperature at 54 °C and the highest pressure of 1.4 bar for a warming time of 09.30–11.00. The efficiency of PTC is 14.2%. It is necessary to measure the insolation directly when heating PTC, so that more accurate efficiency values can be calculated. The apparatus used in this research is illustrated in Figure 4.

In the collectors, the concentration of solar energy must be optimally focused before heat is passed. This collector does so by reflecting or refracting insolation using a mirror or lens to concentrate light. Light is first concentrated before being reflected and focused on one point, and then the energy is passed on to the receiver/absorber [21]. It is necessary to identify the aperture of the concentrator to calculate the amount of insolation received. The insolation received can be calculated directly and does not include reducing some areas resulting from the influence of the incoming angle of the sun or the shadow effect. The insolation entered in the concentrator area will be focused then absorbed by the collector. The energy from insolation is calculated as direct/beam/normal irradiance and is measured using a Pyrheliometer device. For flat-plate collectors, the intensity of insolation calculated is the overall (total) aperture of irradiance measured using a Pyranometer device.

PCMs are one of the heat storage alternatives used as electrical replacement energy in water heating systems [4]. PCM is an effective method by which to improve thermal conductivity and prevent possible environmental interactions or leakage during liquefaction. [22]. The most important difference between PCM and conventional heat storage media such as water or rock is that the melting point of the PCM is within the operating temperature range. Water itself is a PCM, and it is probably the first material to be used, for example, for cooling food with ice. However, because the melting point of water is 0 °C, it cannot be used as a PCM for heat storage applications where the temperature range is above 0 °C. PCM has the following two main characteristics: a very high heat storage density and the ability to store and release large amounts of heat at constant temperatures. This characteristic makes PCM a great alternative to heat storage media for various applications. [23]. In general, the application of PCM is divided into two main groups, namely heat protection and heat storage. The difference is in the heat conductivity of the materials in both applications. Some things to protect heat require a low conductivity value, while a heat storage system can cause a problem as it can expend enough energy but does not have adequate capacity to waste energy rapidly [24].

## 2. Materials and Methods

### 2.1. Beeswax as PCM

The type of PCM used in the study was *Beeswax* (INS No. 901) or yellow beeswax (CAS No. 8006-40-4). These types of PCM have the main component of fatty acid esters of typically 12–14% (ca. 85%) and a long chain of C 24-C 32, fatty alcohol (ca. 1%) with a chain length of C 28-C 35, linear monoesters of wax and hydroxy monoesters (35–45%) with a chain length of generally C 40-C 48, Ester wax complex (15–27%) containing 15-hydroxypalmitic acid, straight hydrocarbon chain (12–16%) with dominant chain length C 27-C 33 [25]. Beeswax melts at 62.28 °C with latent heat between 141.49 kJ/kg (melting) and 145.62 (solidify). The density also changes during melting and solidify which is 789.47 and 819.75 kg/m^3^ respectively. Beeswax was chosen as a thermal energy store in this study because it is suitable for application in a water heater, and local industries also use it. It can be developed as a new sustainable material in thermal energy storage. 

### 2.2. The General Setup of Research

**Figure 5:**
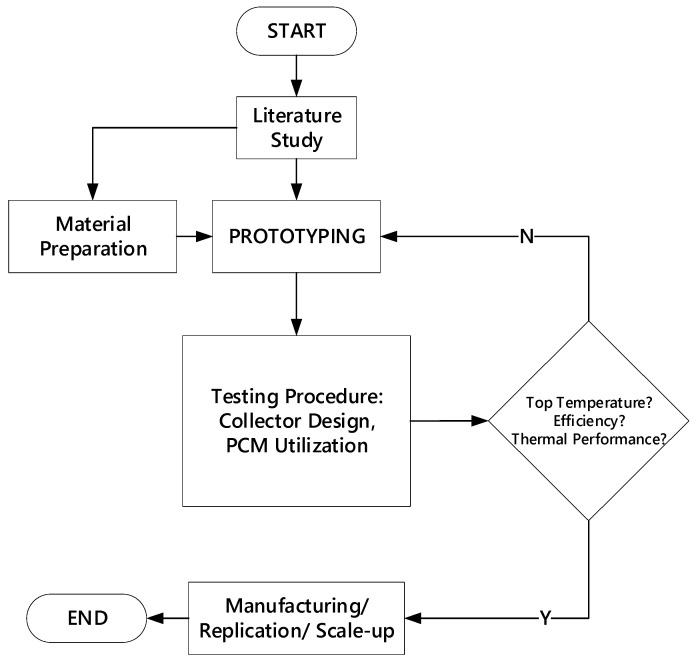
Research Flow Diagram.

This research was conducted experimentally, consisting of several activities, including (i) the manufacturing process of cylindrical-trough-concentrator water heater prototype, (ii) preparation of glass tube/non-glass tube apparatus, (iii) preparation of the hot container embedded with PCM inside the sealed copper tube. This study was delivered through several stages, as shown in Figure 5.

### 2.3. Design and Manufacturing of Solar Water Heaters Prototype

**Figure 6:**
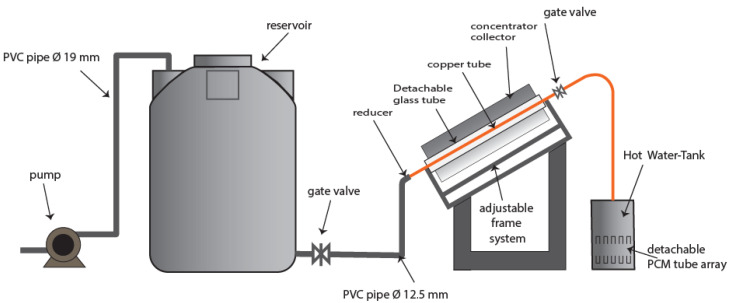
Solar water heater prototype design.

This study used one cylindrical trough collector (CTC) and two variations of the hot water container, namely, the container is equipped with the PCM to maintain the water temperature for as long as possible, and in the second variant, one container is left without a PCM. Typically, this system contains a pump and reservoir with an additional CTC package mounted on an adjustable angle frame of a container. The whole system is represented in Figure 6.

**Figure 7:**
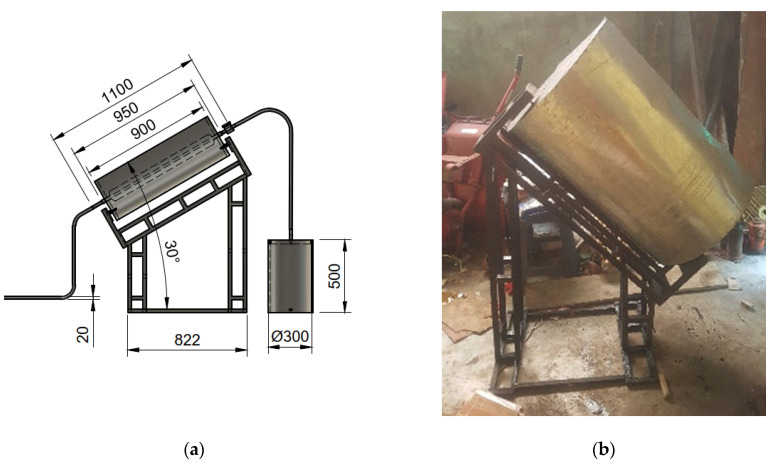
(**a**) design of CTC concentrator collector (**b**) manufactured CTC concentrator collector mounted on an adjustable frame.

The manufacturing process focuses on the water-heater mechanism, including CTC parts embedded with solar reflective film (SRF) affixed to concentrator collector surfaces. These parts are then mounted onto an adjustable angle frame of the concentrator collector support. The result is then presented in Figure 7. 

**Figure 8:**
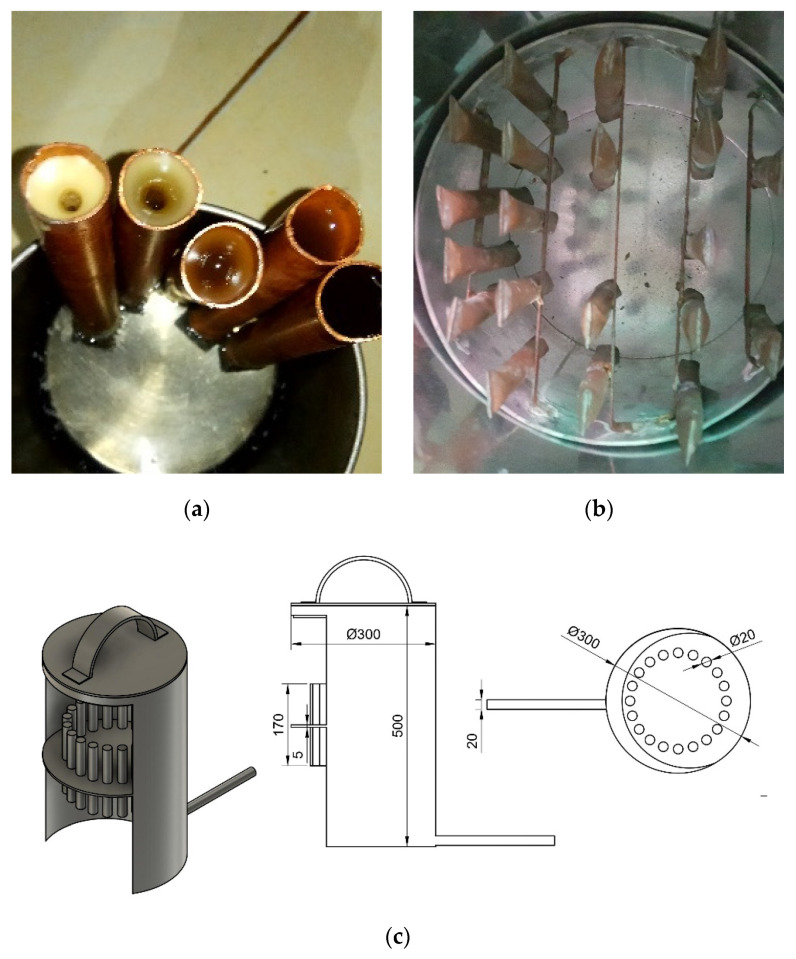
(**a**) beeswax inside the copper tube before sealing. (**b**) sealed copper tube with beeswax inside the water tank. (**c**) detailed dimension of water heater tank equipped with PCM tubes array.

The hot water tank was equipped with a copper tube filled with PCM. The PCM was sealed at both ends; therefore, the beeswax did not leak when the prototype was tested. The copper tube filled with pre-sealed beeswax and the array of PCM-filled copper tubes inside the hot container is shown in Figure 8.

### 2.4. Testing Procedure

This study compared heat release in tanks that use PCM with those that do not use PCM. When the sun’s heat decreased during cloudy day or night, the PCM released the heat absorbed during the day so that the water temperature in the water container remains warm for a little while longer. 

**Figure 9:**
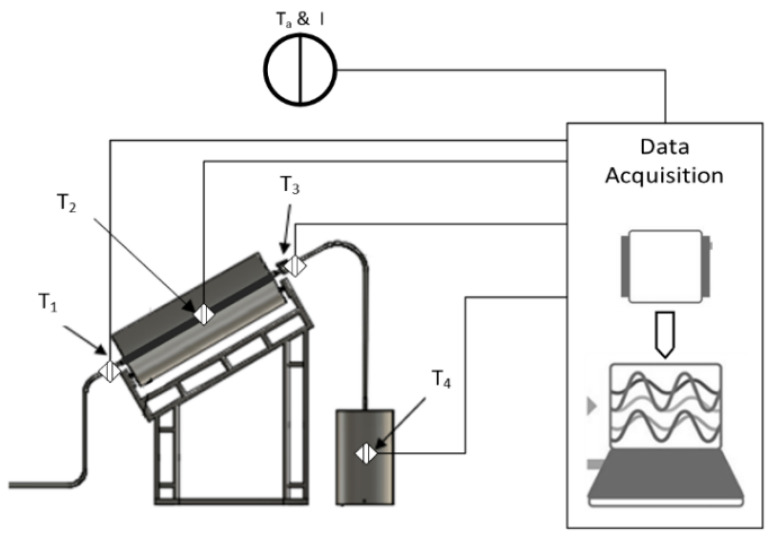
Schematics of sensor placement and data acquisition during the test of solar water heater.

The research was conducted from 08.00 until 17.00 WIB and only conducted during sunny days; therefore, the data obtained were not based on consecutive days or dates. The daily insolation (I) and ambient temperature (T_a_) data were retrieved from an external resource, the US National Renewable Energy Laboratory (NREL), utilizing dedicated software by entering the coordinates of the research site. The coordinates refer to Langsa City, Aceh, Indonesia, located at 4°27.5′ N, 97°56.3′ E. The data retrieved were recorded according to the time of testing. The solar concentrator’s angle was set manually every one hour. The water flow was set up to flow at a constant rate at 1 mL/sec. The temperature data were read with a k-type thermocouple, installed at multiple points, as shown in Figure 9. The four primary k-type thermocouple sensors were mounted in a specific place to the real-time measurement of the initial temperature of the water (T_1_), the focal point on the concentrator (T_2_), the temperature of the water coming out of the solar concentrator (T_3_), and the temperature of the water inside hot water container (T_4_). The data were then consolidated to a data acquisition device, the Agilent 34970A, where data is recorded within every one-minute interval. The data has been represented in a graph in a temperature-time function.

**Figure 10:**
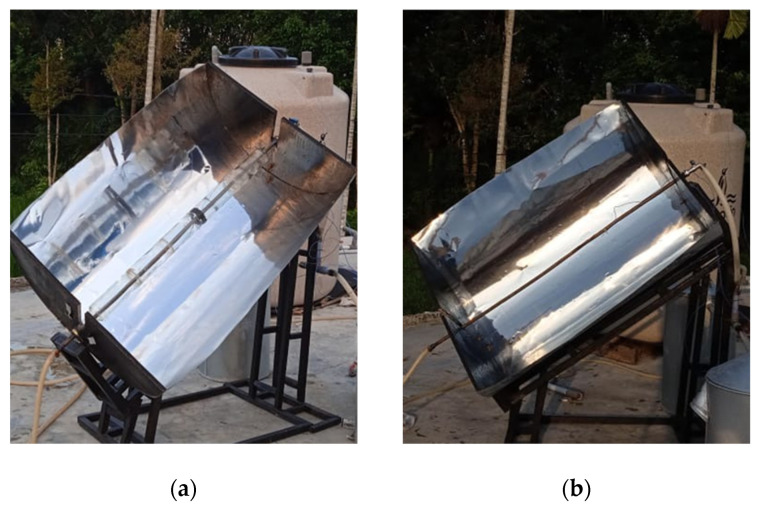
(**a**) solar concentrator in CTC-GT test mode (**b**) solar concentrator in CTC-NGT test mode.

The test also considers the effect of using glass tubes (CTC-GT test mode) and without th use glass tubes (CTC-NGT test mode), with the collector’s angle at 25°, 30°, and 35°. Both types of CTC solar concentrator devices are shown in Figure 10.

### 2.5. The Efficiency of Solar Collector Concentrator

The efficiency compares the amount of energy used to raise the temperature of water flowing in a collector’s pipe and the intensity of insolation that occurs during a given time interval. The average efficiency of solar collectors is the ratio of useful energy to energy captured by the collector. Energy captured by the collector is a function of the collector’s cross-section area multiplied by insolation. The function is briefly expressed as Equation (1).

(1) $$\eta =\frac{Q_{u}}{Q_{abs}}$$

The useful energy was been defined as the portion of final energy available after final conversion to the respective user [26]. For example, electricity becomes light, mechanical energy, or heat in final conversion. The useful-energy in this research refers to the use rate of transferred heat in a fluid, which also means the useful energy used to heat the fluid passing through the collector. The useful heat rate, *Q_u_*, is expressed as Equation (2),

(2) $$Q_{u}=\dot{m}C_{p\;}\left(T_{3}-T_{1}\right)$$

where *ṁ* is the mass of the fluid flow rate (kg/s); *C_p_* is the specific heat of water (kJ/kg. °C); *T*_3_ is the temperature of water discharged from pipe (°C), and *T*_1_ is initial water temperature (°C). Hence, the unit of useful heat rate, which is also defined as useful power, is expressed in watts. 

The amount of energy absorbed by the collector, *Q_abs_*, is calculated in Equation (3).

(3) $$Q_{abs}=A_{c}\cdot I_{r}\;$$

where *A_c_* = collector cross-sectional area (m^2^); and *I_r_* = insolation (W/m^2^). 

The above equations, Equations (2) and (3) are then applied to Equation (1), deriving a ratio of the amount of energy absorbed by the parabolic collector with the energy used by fluid as in Equation (4).

(4) $$\eta =\frac{\dot{m}C_{p\;}\left(T_{3}-T_{1}\right)}{A_{c}.I_{r}}$$

## 3. Results

### 3.1. Hot Water Container without PCM

This subsection explains the comparison of the hot container without PCM. The test was conducted in two tube variations and at predefined angles of the concentrator relative to the sun, as explained in Section 2.5 above. Both CTC-NGT and CTC-GT tests on each angle were conducted on different days from 18 July to 3 August 2021; therefore, the water temperature data in the container obtained was not identical but showed a similar trend. First, the CTC-NGT instrument test was conducted on 18 July 2021, with a collector’s angle of 25 degrees. The research was conducted from 08.00 WIB to 17.00 WIB. At 08.00 WIB, the initial water temperature of the hot container was 22.5 °C, ambient temperature was 29 °C, and solar radiation intensity was 716.24 W/m^2^. 

After the water flowed through a copper pipe, the water temperature in the hot water container rose to 30.5 °C. The rising temperature in this mode is caused by concentrated sunlight transferred directly onto the copper pipes without glass tubes. The focal point temperature was 80.2 °C. Then, the water temperature in the hot water container continued to increase by up to 67.97 °C at 13.00 WIB. The sun’s intensity was 950.6 W/m^2^. After 13.00 WIB, the intensity of solar radiation gradually decreased, causing the water temperature in the hot container to drop until 17:00 WIB. The overall results of the CTC-NGT test at a collector’s angle of 25° show the capability of the system to maintain water temperature above 40 °C for approximately 13 h. The equal indication was also confirmed in other variants of the water heating experiment, and in hot containers without PCM; the data is tabularized as in Table 1 and Table 2. 

**Figure 11:**
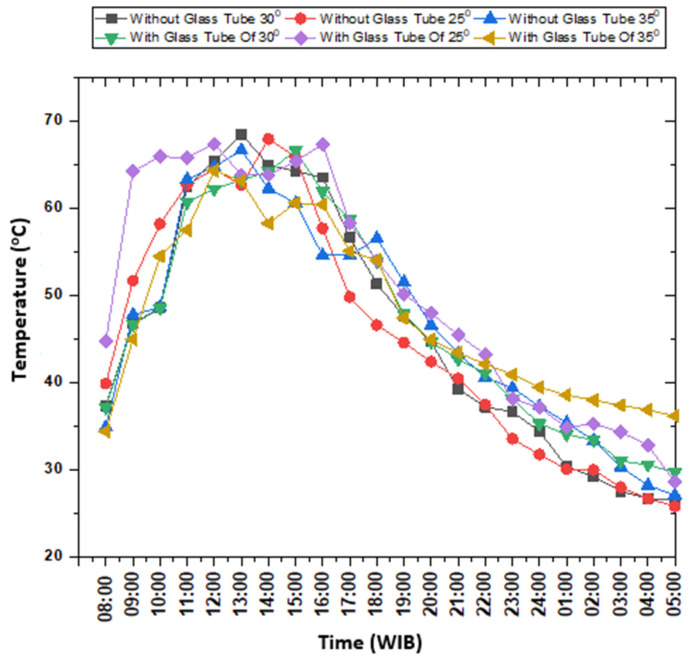
Comparison of hot water temperature inside hot container without PCM during testing solar concentrator in CTC-GT and CTC-NGT mode.

**Figure 12:**
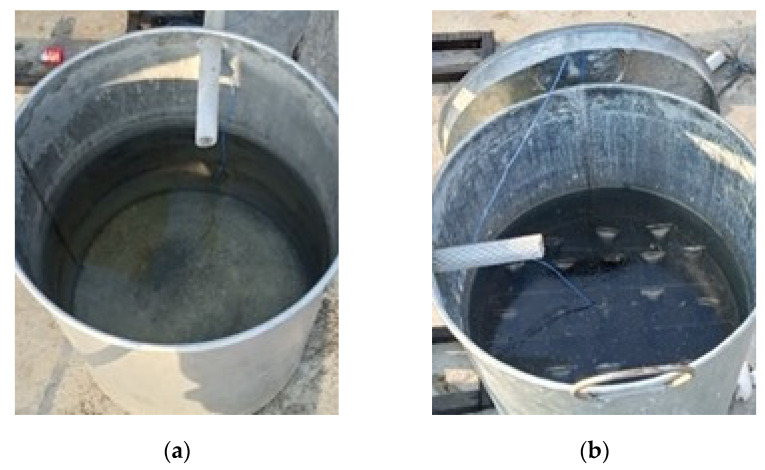
(**a**) hot water container without PCM (**b**) hot water container using PCM.

The data downloaded from the data acquisition device related to temperature measurement in a hot water container without PCM, both in CTC-GT and CTC-NGT mode, are plotted in Figure 11. The graph compares the water temperature in a hot water container without PCM at a collector’s angle of 25, 30, and 35 degrees, respectively. The hot container with PCM and without PCM is shown in Figure 12.

### 3.2. Hot Water Container with PCM

This subsection explains the comparison of the hot container with the use of PCM. The test is still conducted in both CTC-GT and CTC-NGT mode, with an angle relative to the sun 20°, 30°, and 35°.

The experiment measuring water temperature in hot containers embedded with PCM also demonstrated the same phenomenon as explained in the previous section in the hot container without PCM. Nevertheless, the temperature release is somewhat slower. The results of the experiment are shown in Table 3 and Table 4.

**Figure 13:**
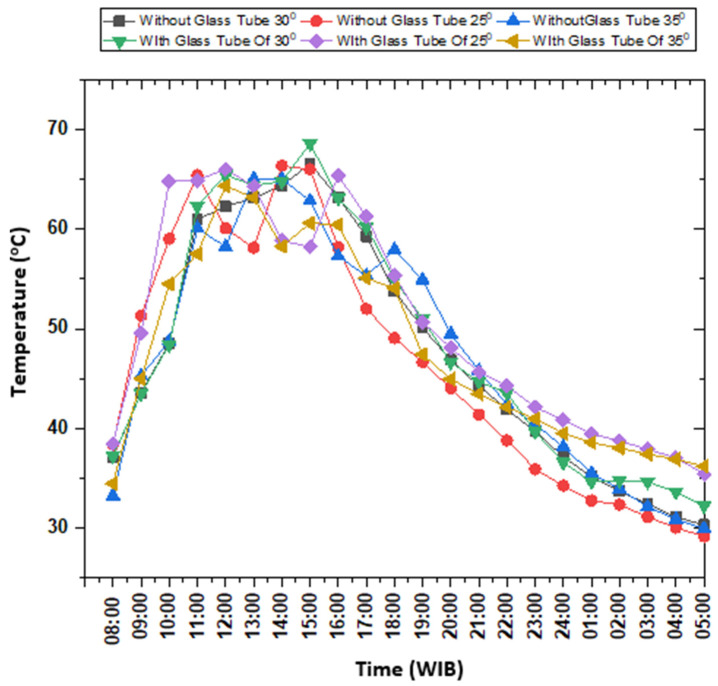
Water temperature comparison during testing in CTC-GT and CTC-NGT mode.

The data downloaded from the data acquisition device concerning this experiment of hot water container with PCM is plotted as shown in Figure 13.

The figure shows a water temperature in the hot container during CTC-NGT and CTC-GT tests at the collector’s angle of 25°, 30°, and 35°. In principle, the method of retrieving test data is the same as the discussion explained in the subsection of the hot water reservoir without PCM. This discussion confers (i) the effect of beeswax on the temperature of hot water obtained, (ii) the time of heat stored in hot water for 24 h, and (iii) a comparison of the instrument’s performance without using beeswax.

Using the equation provided in Section 2.5, the efficiency of the cylindrical trough concentrator in both test modes is calculated. At the given collector’s angle, CTC-NGT test mode shows the efficiency of 30.7%, 31.9%, and 31.7% correspondingly, while CTC-GT mode provided 27%, 29.9%, and 29.6%. The calculation result is elaborated in Table 5.

## 4. Discussion

The research concerning the use of a PCM in a hot water container of a water heater system was conducted. Prototype testing was conducted from 18 July to 3 August 2021, and testing was separated into two parts, the first conducted during daylight, from 08:00 to 18:00 WIB, which aimed to observe the energy conversion process from the sun into heat and store it in the water. The second part was conducted after the sun began to set, which means the sun’s energy gradually diminished, so as to observe the process of energy released from water. The results showed that water heaters using concentrated solar power systems could produce 16 L of hot water and retain temperature at a range of 40–60 °C for 4 h. With the addition of beeswax, the water temperature was maintained at 40–60 °C for 5 h, with the same capacity. This suggested that the performance of the parabolic trough water heating system equipped with beeswax is excellent. The system maintained the heat after reaching a temperature above 40 °C and provided a sufficient amount of 60 L of hot water per day.

In terms of the utilization of the glass tube, however, in the CTC-NGT mode, the copper tube can directly receive the sunlight to heat the water and shows better efficiency. Nevertheless, some of the energy received quickly dissipates due to the convection loss. The average value of advantageous or usable energy received by CTC-GT at the collector’s angle of 25°, 30°^,^ and 35° is 130.13 W, 145.51 W, and 108.41 W, respectively. Alternatively, the CTC-NGT test mode at the same angle was calculated as 154,46 W, 148,92 W, and 146.18 W, respectively. The average efficiency of the CTC-GT test mode at a collector’s angle of 25°, 30°, and 35° is 30.7%, 31.9%, and 31.7%, while CTC-GT mode provided 27%, 29.9%, and 29.6%.

Several of the findings in this study are significant. The water-flow rate in the collector affects the temperature of the water coming out of the collector; the slower the flow, the higher the water temperature. The collector’s angle relative to the sun dramatically affects the total insolation captured by the collector; the smaller the angle, the more insolation is captured by the concentrator. Moreover, glass tubes seem to reduce the energy dissipating from the concentrator collector.

Overall, this research demonstrated a promising result, that the utilization of PCM has increases the time during which hot containers are able to retain temperature after the sun is set. We also found that the whole water heater design with an embedded PCM should be adapted into a more appropriate design and using the outlined manufacturing process to achieve better performance. The technology proposed in this research can also be modified or upgraded into a hybrid setup, combining solar and biomass energy. Since the region is provided with abundant biomass or lignocellulosic-based energy sources, this will promote a more economical approach to energy utilization [27,28]. Nevertheless, hybrid technology adoption will lead to a slight to moderate modification of the currently developed technology. As for wind energy integration, there will be a comprehensive adjustment to the development of the proposed methods, since they cannot be directly used and require a relatively expensive conversion step.

## Figures and Tables

**Table 1:** CTC-NGT test results at angles of 25°, 30°, and 35° in the container without PCM.

Collector’s Angle	Date	Time (WIB)	T_1_ (°C)	T_2_ (°C)	T_a_ (°C)	I (W/m^2^)	Time T_Ᾱ_ > 40 °C (Hour)
25	18/7	08.00	22.5	30.5	29	716.24	13
		12.00	29.5	64.8	39	994.6	
30	24/7	08.00	18.7	32.3	29.7	706.24	12
		13.00	26	67.39	39.7	998.48	
35	26/7	08.00	23.2	33.7	28.5	710.24	13
		13.00	28.7	64	33.5	995.26	

**Table 2:** CTC-GT test results at angles of 25°, 30°, and 35° in the container without PCM.

Collector’s Angle	Date	Time (WIB)	T1 (°C)	T2 (°C)	Ta (°C)	I (W/m^2^)	Time TᾹ > 40 °C (Hour)
25	28/7	08:00	18.7	32.2	28.2	702.16	15
		12:00	30	67.3	38.7	997.88	
30	31/7	08:00	19.5	32.2	29.7	716.46	14
		13:00	29.5	67.8	42.2	999.05	
35	3/8	08:00	26.5	34.5	27.5	626.34	14
		13:00	29.5	73.5	39	1026.4	

**Table 3:** CTC-NGT test results at angles of 25°, 30°, and 35° in the container with PCM.

Collector’s Angle	Date	Time (WIB)	T1 (°C)	T2 (°C)	Ta (°C)	I (W/m^2^)	Time TᾹ > 40 °C (Hour)
25	18/7	08:00	22.5	29	29	716.24	13
		12:00	29.5	62.7	39	994.6	
30	24/7	08:00	18.7	26.2	29.7	706.24	12
		13:00	26	72.3	39.7	998.48	
35	26/7	08:00	23.2	32	28.5	710.24	13
		13:00	28.7	58	33.5	995.26	

**Table 4:** CTC-GT test results at angles of 25°, 30°, and 35° in the container with PCM.

Collector’s Angle	Date	Time (WIB)	T1 (°C)	T2 (°C)	Ta (°C)	I (W/m^2^)	Time TᾹ > 40 °C (Hour)
25	28/7	08:00	18.7	33.2	28.2	702.16	15
		12:00	30	66.8	38.7	997.88	
30	31/7	08:00	19.5	32.2	29.7	716.46	14
		13:00	29.5	68	42.2	999.05	
35	3/8	08:00	26.5	34.5	27.5	626.34	14
		13:00	29.5	65	39	1026.4	

**Table 5:** The efficiency of the solar concentrator at a given angle of each test.

Test Mode	Collector’s Angle n(°)	Useful Power (Watt)	Efficiency (%)
CTC-NGT	25	154.46	30.7
CTC-GT	25	130.13	27
CTC-NGT	30	148.92	31.9
CTC-GT	30	145.51	29.9
CTC-NGT	35	146.18	31.7
CTC-GT	35	108.41	29.6
